# How to Influence Rural Tourism Intention by Risk Knowledge during COVID-19 Containment in China: Mediating Role of Risk Perception and Attitude

**DOI:** 10.3390/ijerph17103514

**Published:** 2020-05-18

**Authors:** Hui Zhu, Fumin Deng

**Affiliations:** Business School, Sichuan University, Chengdu 610065, China; 2015225020124@stu.scu.edu.cn

**Keywords:** rural tourism, risk knowledge, tourism risk perception, risk aversion attitude, knowledge-attitude-behavior (KAB)

## Abstract

With both cost and safety taken into account in the context of the life-threatening COVID-19 pandemic globally, rural tourism is expected to be the top choice for Chinese residents for relaxation and enhancing parent-child relationships. In this paper, a structural equation (SEM) model was proposed to compare risk knowledge, risk perception, risk aversion attitudes and behavioral intentions towards rural tourism. According to the empirical results, there was a large proportion of tourists showing preference for rural tourism recently. Potential participants in rural tourism paid most attention to the performance realization and time cost of scenic spots, while the psycho-social risk posed by COVID-19 had little impact. The inherent risk nature of risk aversion attitudes made knowledge of the pneumonia risk less effective in reducing tourists’ intentions, while knowledge of the pneumonia risk was more effective in alleviating the risk perception that potential tourists have towards rural tourism. With regard to travel intention and recommendation intention of rural tourism, the negative impacts of risk aversion attitude were more considerable compared to risk perception. Meanwhile, the parallel mediating effect of risk perception and risk aversion attitude in rural tourism needed to be taken into consideration together.

## 1. Introduction

In 2020, the global outbreak of COVID-19 has made an enormous impact on a wide variety of different industries. The slump in outbound expenditure has caused a severe damage to such services as transport, tourism, catering, retail and entertainment. It is estimated by the World Tourism and Travel Council (WTTC) that COVID-19 will cause the global tourism industry a huge loss that amounts to a minimum of 22 billion dollars [1]. In China, the immediate loss suffered by the tourism industry as a whole during the Spring Festival in 2020 will hit 550 billion yuan. According to the China tourism academy, the number of domestic tourists will drop by 56% and 15.5% in the first quarter and the whole year of 2020, respectively. The revenue made by the domestic tourism industry also shrank by 69 percent and 20.6 percent respectively, which means a reduction of revenue of 1.18 trillion yuan [2]. COVID-19 definitely caused a major tourism crisis [3].

**Figure 1:**
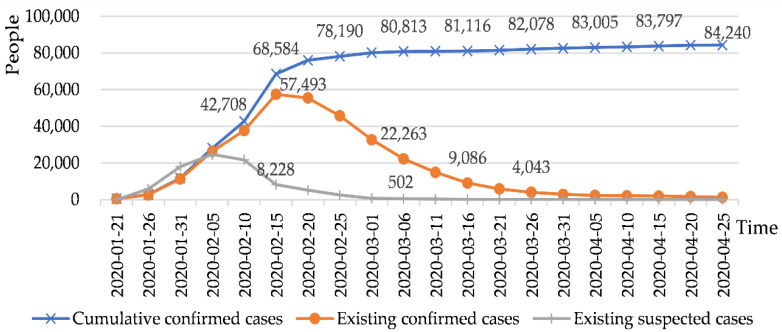
Distribution of COVID-19 cases in China. Note 1. Data was collected from Wind database.

**Figure 2:**
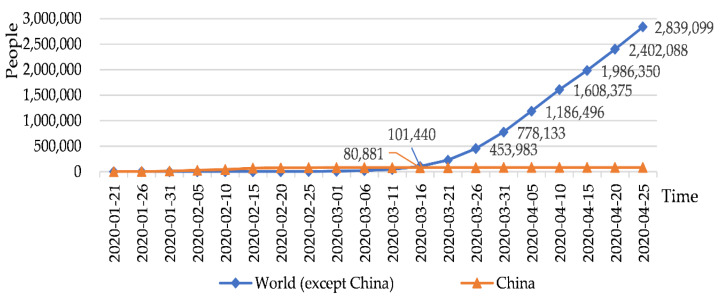
Confirmed cases of COVID-19 in world. Note 1. Data was collected from Wind database.

China was the first to be hit with the COVID-19 outbreak, which left most Chinese citizens in self-isolation starting at the end of January, 2020. In China, the growth rate of confirmed COVID-19 cases has declined in late February, 2020 (Figure 1). By contrast, the number of confirmed foreign cases has surpassed that of domestic cases since 15 March 2020 (Figure 2). After various containment measures were put in place across China, the pandemic was brought under control effectively, which also prompted a strong desire among many Chinese residents to go outside. What kind of policy can satisfy the desire to go out and ensure safety to a certain extent? Rural tourism might be the first choice. As a significant tool for rural development [4], rural tourism shows such advantages as low travel cost [5], short time consumption and low flow density, which could fulfill the psychological requirements of people to enjoy natural scenery and be guaranteed safety while going out. Meanwhile, the longer shutdown period of travel agencies, airlines and hotels makes people shift their attention to rural tourism for meeting their outdoor needs after the COVID 19 pandemic has been brought under control to some extent, thus presenting a valuable opportunity for the rural tourism economy to recover rapidly.

As argued by Li [6], risk perception is the starting point for the judgment of crisis impact on the tourism market. Due to the public health emergency, the behavior and preference of people are subject to a considerable influence from perceived risk. There are some scholars even finding out that perceived risk better accounts for tourism consumption behavior than perceived value [7,8]. Currently, study in this regard is narrowly focused on the effect of emergencies on the tourism industry as a whole and on overall travel intention, which ignores the analysis of different types of tourism and people’s preferences in this exceptional time. Firstly, the impact of COVID-19 is far more significant compared to SARS which broke out back in 2003. As can be seen by the strongest persistent prevention of the epidemic, Chinese people have acquired mature and accurate knowledge about how to deal with the outbreak of epidemic. Therefore, the impact of the epidemic can be better highlighted by taking Chinese residents as the research object. Secondly, the primary motivation to promote the willingness that people have travel stems from the needs and purposes of travel, as argued by the push-pull theory [9]. After a period of hard work, people will take the initiative to have relaxation. However, there are other influencing factors on whether they would take action. Therefore, this paper is limited to conducting investigation into behavioral intention. Thirdly, the Chinese government has announced a full resumption of production. The natural scenery of rural tourism is effective in facilitating relaxation, reducing the stress caused by the outbreak of epidemic, and enhancing parent-child relationships. In comparison with such indoor activities as museums, science and technology museums [10] and tourist attractions, the density of tourist flow brought about by rural tourism is clearly lower, as a result of which the perception of physical risks diminishes to some degree. 

This study aims to determine the relative willingness of people under the context of epidemic to engage in rural tourism and to identify the factors they would take into consideration when rural tourism is chosen. This paper makes three innovations. Firstly, the perspective of perceived risk was more convincing in studying travel intention rather than perceived quality and perceived usefulness, due to the characteristics of epidemic emergencies. Secondly, as opposed to taking the whole tourism industry as the research object, this paper related avoidance behavior theory to the rural tourism scene to analyze tourists’ relative travel intentions and the internal factors affecting tourists’ rural tourism intentions. Thirdly, the knowledge-attitude-behavior (KAB) model was introduced into the tourism research field, and the research model of this paper was constructed around risk perception. In this regard, this paper drew a comparison of rural tourism knowledge and pneumonia risk knowledge, as well as of rural tourism risk perception and risk aversion, by combining the risk knowledge-risk perception-behavior intention and risk knowledge- risk aversion attitude-behavior intention models, based on which was identified as the major influencing factor in rural tourism and travel. As it is unlikely for tourists to accept tourism risk factors with ease [11], tourism managers need to give careful consideration to risk perception factors and to make a better plan for the development of tourism [12,13]. Not only do the research results provide reference for the preparation of rural tourism during the epidemic period, they also reveal the travel focuses of tourists regarding other tourism enterprises.

## 2. Literature Review and Research Framework

### 2.1. Tourism Risk Perception

The theory of perceived risk was first suggested by Bauer [14], who raised the notion of risk for marketing and applied it to account for consumer behavior. Mouwen and Minor [15] defined perceived risk as the probability for negative results to play out. Based on this, tourism risk perception was regarded as the subjective judgment made by consumers that leads to negative results for tourism [16,17], which resulted from the asymmetry objectively existent in tourism safety information and of the subjective perception that tourists have [18]. After the impact of sudden crisis events, this will increase in a short time [19]. People with different personal characteristics also have different perceived risks to the same form of travel [20]. According to Kozak [21], older people with travel experience had less awareness of potential risks such as health risks, terrorism or natural disasters. Hwang and Lee specialized in the perception of tourism by the elderly [22,23,24,25], and they pointed out the different and higher requirement of guide services [22] and customer-customer rapport [24] during senior tourism. Lepp and Gibson [26] believed that women have a high perception of health and food risks, and international tourists with rich travel experience may have a low perception of risks. Yao and Hou [27] analyzed the dimension of women’s perceived risk from the perspective of sex. Therefore, the sample distribution in the study deserves our attention and analysis.

Tourism risks include time risk, satisfaction risk, psychological risk [28], social risk, physiological risk, security risk and capital risk [18,29,30]. In empirical studies, tourism risk perception has been identified as multi-dimensional. Stone and Gr O Nhaug [31] conducted a study to verify the existence of six risk dimensions based on Kaplan and Jacoby’s [32] research, including financial risk and functional risk, which received recognition in the subsequent period. By exemplifying the impact of earthquake events on tourists, Zhu et al. [33] argued that tourism risk perceptions involve functional risk, crisis risk and culture conflict risk, with the functional risk perception of consumers being the highest. As indicated by Xu et al. [34], apart from the aforementioned six dimensions of physical risk, functional risk, financial risk, communication risk, psychological risk and social risk, service risk and equipment risk are also included in the perceived risk of tourism consumers. By considering tourists’ travel risk perception under the haze weather, Zhang and Yu [35] categorized tourism risk perception into physical risk, functional risk, psychological risk, and cost risk. In Table 1, a summary was made of the relevant studies on how the tourism risk perception dimension is defined with reference to Tsaur et al. [36], Chen et al. [37] and Yao and Hou [27].

As shown in Table 1, with regard to tourism risk perception, such dimensions as performance risk, physical risk, psychological risk, and social risk are regarded as relatively significant. In many studies, an analysis was conducted of financial risk, time risk and equipment risk. As financial risk and time risk can be classed as cost risk [35], the seven dimensions shown in Table 1 are converted into six dimensions, including performance risk, physical risk, psychological risk, social risk, cost risk and equipment risk. This group also constitutes tourism risk perception involved in the model of this paper.

### 2.2. Impact of Risk Knowledge on Risk Perception

Given the perception of risk mainly stems from the uncertainty of events, Lepp and Gibson [26] indicated the possibility that those international tourists with plenty of tourism experience and sufficient risk knowledge could perceive less risks. The tourists’ risk perception of the crisis will increase significantly if the emergency is new or unknown to tourists. With earthquake risk perception exemplified, Li [6] discovered the significantly negative impact of earthquake knowledge on earthquake risk perception, which suggests that tourists’ risk perception would be reduced with increasing awareness of risk knowledge. By means of multiple linear regression, Chai et al. [53] verified the close association between risk knowledge and risk perception. In other fields, Wang and Xu [54] analyzed the impact of interest involvement and information saturation on the public perception of risk, showing that the mastery of risk-related knowledge negatively affects the public perception of risk. Using typology tools, Liu [55] verified the “knowledge weakening hypothesis of public risk perception”, and that risk knowledge had negative influence on public risk perception. Wang et al. [56] studied the factors influencing consumers’ risk perception of food additives and found that consumers’ awareness of food additives had the most significant negative effect on their risk perception. The outbreak of COVID-19 has been declared a public health emergency. For the impact of the epidemic on the behavioral intention towards rural tourism to be highlighted, tourism risk knowledge and pneumonia risk knowledge were chosen as the dependent variables in this paper to conduct comparative analysis. Based on the negative relationship between risk knowledge and perceived risk, the following hypotheses are proposed:

**Hypothesis** **1** **(H1).** *Tourism risk knowledge negatively and significantly affects risk perception of rural tourism.*

**Hypothesis** **2** **(H2).** *Pneumonia risk knowledge negatively and significantly affects risk perception of rural tourism.*

### 2.3. Impact of Risk Knowledge on Risk Aversion Attitude

Most of the existing research focuses on the influence of universality, professional scientific knowledge or tacit knowledge, and local knowledge on risk perception or acceptance [55]. Zhao [57] divided knowledge into three categories: social information (obtained by interacting with the outside world), major-oriented knowledge (obtained through the study of specialized courses and related to a specific learning field of knowledge information) and general knowledge (access to information by foraging, not limited to a specific area). For most ordinary residents, risk knowledge mainly comes from social information and general information.

As the understanding of medical knowledge can reduce the uncertainty caused by a disease, there is a positive correlation between knowledge and aversion attitude [58,59]. However, in other fields, we found a negative correlation between risk knowledge and risk aversion attitude. In terms of nuclear power popularization, the higher the level of subjective knowledge, the higher the acceptance of nuclear power risks [60,61], and the lower the willingness to evade. In the financial scenario, financial knowledge was positively correlated with risk preference attitude, and risk preference attitude positively affected financial market participation [62,63,64]. Risk preference attitude was a significant mediating variable. Zhao [57] found that general risk knowledge from social interaction would promote willingness to take risks; that is, the more information, the more individuals would prefer to take risks.

This difference was caused by the different benefit and cost structure of behavior. Disease risk in the medical and health field is directly related to the individual’s illness and health, so the cost proportion of the risk is much higher than the benefit. On the other hand, in financial investment and outbound tourism, the cost of taking investment/travel risks is lower than the benefits, so the mastery of risk knowledge can increase the acceptance of risks. When affected by risks, people are inclined to act in a rational way and mitigate potential risks by either complete or incomplete risk avoidance [65]. In reality, there are more consumers preferring incomplete avoidance to minimize the foreseeable losses while receiving the benefits created by the behavior [66], as manifested in the search for confirmation information in advance [65], the formulation of a response plan, and prompt choice of travel time and travel method based on risk characteristics [21]. From the perspective of consumers, their decision making and risk judgment are subject to influence from tourism risk perception [67,68]. In this paper, rural tourism was viewed as an incomplete avoidance choice in relation to travel.

Based on the characteristics of risk knowledge and risk attitude towards rural tourism, an assumption was made in this paper that tourists will take a receptive attitude towards tourism risk and reduce the tendency of risk avoidance after acquiring more risk knowledge. In order for risk perception to be matched, risk aversion attitude was selected as the mediating variable in this paper, based on which the following hypotheses were proposed:

**Hypothesis** **3** **(H3).** *Tourism risk knowledge negatively and significantly affects risk aversion attitude.*

**Hypothesis** **4** **(H4).** *Pneumonia risk knowledge negatively and significantly affects risk aversion attitude.*

### 2.4. Impact of Risk Perception on Behavioral Intention

Tourism risk perception could have a considerable impact on the decisions made by tourists [20]. The overall level of tourism risk perception can be improved by risk perception in a dimension [69]. A lower possibility of potential tourists [70,71] can contribute to the higher probability of consumers reducing risks through risk aversion behavior. Most studies have focused on the influence of service quality on tourism intention. Transportation convenience, tourism safety, accommodation convenience, the degree of tourism information, travel agency services, leisure time and conformity psychology were all positively correlated with tourism intention [72,73,74]. The above factors can be converted into risk perception factors and the positive relationship with tourism intention changed to negative. Hua et al. [75] proved that safety concerns, geographical damage, casualties and damage to facilities and equipment, psychological taboo, ethical conflicts, cost concerns and tourism intention had a direct negative correlation. Guo et al. [76] considered the negative impacts of social risk, political risk, and culture risk on tourism intention in Japan. Luo and Zhu [77] analyzed influence factors on willingness to use balance treasure, and the results showed that perceived ease of use, perceived usefulness, perceived benefits, subjective norms, and perceived behavior control affect the users positively. But security risk, economic risk and time risk had a significant negative impact on users’ willingness. Due to the ongoing outbreak globally, people are still banned from getting out much, so the impact of COVID-19 on tourist behavioral intention was analyzed in this paper. The following hypotheses are proposed:

**Hypothesis** **5** **(H5).** *Rural tourism risk perception negatively and significantly affects travel intention in rural tourism.*

**Hypothesis** **6** **(H6).** *Rural tourism risk perception negatively and significantly affects recommendation intention in rural tourism.*

**Hypothesis** **7** **(H7).** *Rural tourism risk perception plays an intermediary role in the relationship between tourism risk knowledge and rural tourism travel intention.*

**Hypothesis** **8** **(H8).** *Rural tourism risk perception plays an intermediary role in the relationship between tourism risk knowledge and rural tourism recommendation intention.*

**Hypothesis** **9** **(H9).** *Rural tourism risk perception plays an intermediary role in the relationship between pneumonia risk knowledge and rural tourism travel intention.*

**Hypothesis** **10** **(H10).** *Rural tourism risk perception plays an intermediary role in the relationship between pneumonia risk knowledge and rural tourism recommendation intention.*

### 2.5. Risk Knowledge—Risk Aversion Attitude—Behavioral Intention Model

Risk attitude was defined as “consumers’ consistent choice tendency to face different risk levels” or “consumers’ willingness to accept risks” [78], and is an inherent risk selection attribute [79]. Nevertheless, Weber et al. [80] argued that the attitude taken by consumers towards risks is determined by their expected benefits and perceived risks, while perceived risks bear a negative correlation with their risk attitudes. When accounting for specific consumer behaviors, Pennings [81] discovered the differences in risk acceptance among individuals. Therefore, it is considered insufficient for perceived risk alone to back the results. For risk-averse people, a slight increase in perceived risk can lead to a change in their buying behavior. Nevertheless, for risk takers, it is possible that a sufficiently high perceived risk has no impact on their buying behavior [82]. Therefore, risk perception and risk attitude can exert influence on behavioral intention at the same time.

Compared with risk seekers, risk-neutral and risk-averse people were more likely to choose vaccination to avoid the risk of hepatitis B [83]. The research of Lola and Gregg [84] showed that the difference of individual risk attitudes will directly affect decision-making cognition: risk-seekers focus more on good results, while risk-avoiders focus more on bad results. Financial knowledge was positively correlated with stock market and financial market participation, while risk-averse people had lower stock market and financial market participation [85]. Therefore, in the rural tourism scene, risk avoiders will pay more attention to the risk consequences of travel, and risk avoiders are less willing to travel. The relationship between risk aversion attitude and rural tourism intention should be negative.

The structure of “risk knowledge-risk perception-behavioral intention” established in this paper was compared using “Knowledge-attitude-behavior” (KAB), a mature model which splits the change in people’s behavior into three continuous processes: acquiring knowledge, generating belief and forming behavior [86]. Unlike other theoretical models of consumer behavior, the KAB model is mainly purposed to explore the correlation between knowledge, attitude and behavior. With regard to other theoretical models of consumer behavior, for example, the planned behavior theoretical model, knowledge is treated as an external variable that could exert influence on attitude. When it comes to consumer behavior, attitudes, subjective norms and perceived behavior control are considered to be the influencing factors. In the theoretical model of planned behavior, however, knowledge variables are not the focus of research. In this paper, our focus was placed on how risk knowledge influences behavioral intention, for which the KAB model was chosen.

At present, KAB theory has been met with widespread application in clinical medicine, education and public health. By constructing a model of Chinese universities’ sexual knowledge attitudes-related behaviors, Zhang et al. [58] found out that a neutral attitude exerted a partial mediating effect on how influential sexual health knowledge would be on sex-related behaviors. By applying the structural equation model, Zeng et al. [59] validated the positive direct impact of knowledge, attitude and behavior as well as the indirect impact of knowledge on behavior in hypertension. Therefore, it can be judged that attitude can be treated as a mediating variable in the association between knowledge and behavior. At present, the KAP theory has yet to be applied in the field of tourism. Therefore, based on the KAB theory and the characteristics of the rural tourism scene, this paper proposes the following hypotheses:

**Hypothesis** **11** **(H11).** *Risk aversion attitude negatively and significantly affects travel intention in rural tourism.*

**Hypothesis** **12** **(H12).** *Risk aversion attitude negatively and significantly affects recommendation intention in rural tourism.*

**Hypothesis** **13** **(H13).** *Risk aversion plays an intermediary role in the relationship between tourism risk knowledge and rural tourism travel intention.*

**Hypothesis** **14** **(H14).** *Risk aversion plays an intermediary role in the relationship between tourism risk knowledge and rural tourism recommendation intention.*

**Hypothesis** **15** **(H15).** *Risk aversion plays an intermediary role in the relationship between pneumonia risk knowledge and travel intention in rural tourism.*

**Hypothesis** **16** **(H16).** *Risk aversion plays an intermediary role in the relationship between pneumonia risk knowledge and rural tourism recommendation intention.*

### 2.6. Other Influence Factors on Behavioral Intention

Although risk perception includes many factors that influence tourists’ behavioral intention, there are differences in the influencing factors in different tourism scenarios. With regard to domestic and overseas tourism, such perceived risks as the political situation, natural disasters, public health and personal safety are taken into account [42,46,50,65,87,88]. Tomas and Jiri [87] analyzed the political security risk on an overseas tour. Wu [88] concluded that cultural conflict is one of the factors affecting tourists’ choice of outbound travel. Individual characteristics of tourists will also lead to differences in consumer behavior. Mitchell and Vassos [8] analyzed the differences in perceived risk under different cultures and gender. This paper concentrated on the rural tourism scene close to the area where the interviewees reside. They were required to familiarize themselves with local policies and cultural customs. Therefore, such factors as political risk and cultural conflict were not considered for the research model. Descriptive statistical analysis was conducted to explain the personal information.

As one of the sources of risk knowledge, media opinions and government-oriented policies will also have an influence on consumer behavior to some extent. When Huang and Jennifer took Taiwan as an example to study earthquake damage and tourism recovery, they found that media reports on the damage brought by the earthquake to Taiwan island affected potential tourists [89]. Milo and Yoder found that media coverage after the disaster reduced the willingness of tourists or potential tourists to travel, which further complicated the post-disaster recovery [90,91]. State media reports in China took a dominant position in the dissemination of information under the guidance of the state government. Meanwhile, it was believed that Chinese residents had acquired relatively sufficient and correct knowledge about the risks associated with the two months of self-isolation through participation in the prevention and control of the Covid-19 pandemic. Therefore, media reports were excluded from the model as an independent variable.

## 3. Questionnaire Design and Descriptive Statistics

### 3.1. Questionnaire Design

After literature review, risk perception (PCP) was mainly assessed from six different perspectives, which are physical risk (PHY), equipment risk (EQU), cost risk (COS), psychological risk (PSY), social risk (SOC) and performance risk (PER). In this paper, both risk perception and risk aversion attitude were treated as the mediating variables to investigate how the willingness of participating in rural tourism could be affected by the risk knowledge acquired by potential tourists.

In order for data collection, the questionnaire survey method of the 5-level Richter scale was applied [18]. The design of questionnaire items was premised on the current literature [92] and was combined with the characteristics of rural tourism. This final questionnaire item is comprised of two parts. The first conducted investigation into tourism risk perception, risk aversion, risk knowledge and rural tourism intention, while the other collected demographic information [93,94]. In the questionnaire, three and four questions were raised respectively for physical risk (PHY), equipment risk (EQU) and psychological risk (PSY), mainly from Han [43], Yao and Hou [27], Xu et al. [34], and Zhang and Yu [35]. Performance risk (PER), cost risk (COS) and risk aversion attitude (ATT) were respectively established with four items, with major reference to the questionnaire items of Zhang and Yu [35], Yao and Hou [27], Xu et al. [34] and Liu et al. [83]. The three items of social risk (SOC) were suggested by Han [43]. The questions on tourism risk knowledge (KT) and pneumonia risk knowledge (KP) were premised on Li’s [6] and Xu’s [52] analysis of event characteristics, with five and six questions respectively. There were six items involved to assess the behavioral intention of rural tourism, including three travel intention (TI) items and three recommended intention (RI) items. Based on the research conducted by Xu et al. [52], Lai and Chen [95], and Zhao et al. [96], the subject content was adjusted. The specific questionnaire items are listed in Table 2.

### 3.2. Data Collection Method

With such advantages as high accessibility, low cost and ease of operation, the network questionnaire can be issued to conduct surveys that cannot be completed in the form of probability sampling [97]. During the combat against COVID-19 epidemic in China, both accidental sampling and snowball sampling methods were applied to collect questionnaire data while avoiding contact with people of all ages and occupations. Respondent-Driven Sampling was conducted, which reduced sample bias by requiring the respondents to recommend a specific number of peer groups [98]. In order to prevent the impact of sample size, a percentage of data was used to analyze the grouped samples. As for the presentation of the questionnaire, the questions, options and check boxes of each group of questions were presented all together for the interviewees to fill in. The background regarding epidemic situation, going outside, rural tourism and purpose of the investigation were thoroughly explained. Questionnaire items like “Is there a covid-19 outbreak in your province?”, “In the near future, what do you think of the possibility of the following tourism risks happening in the countryside?” and “Please select the type of travel risk mentioned above.” were also listed to collect valid samples. In addition, a check was conducted on the time taken by the questionnaire fillers, IP address, whether the same option was repeated several times and other information to control the quality of data. There were totally 474 questionnaires distributed online by means of WeChat QR code and network link. 412 valid questionnaires recovered finally, with the effective recovery rate reaching 86.7%.

### 3.3. Descriptive Statistical Analysis

#### 3.3.1. Demographic Information and Basic Viewpoints

**Figure 3:**
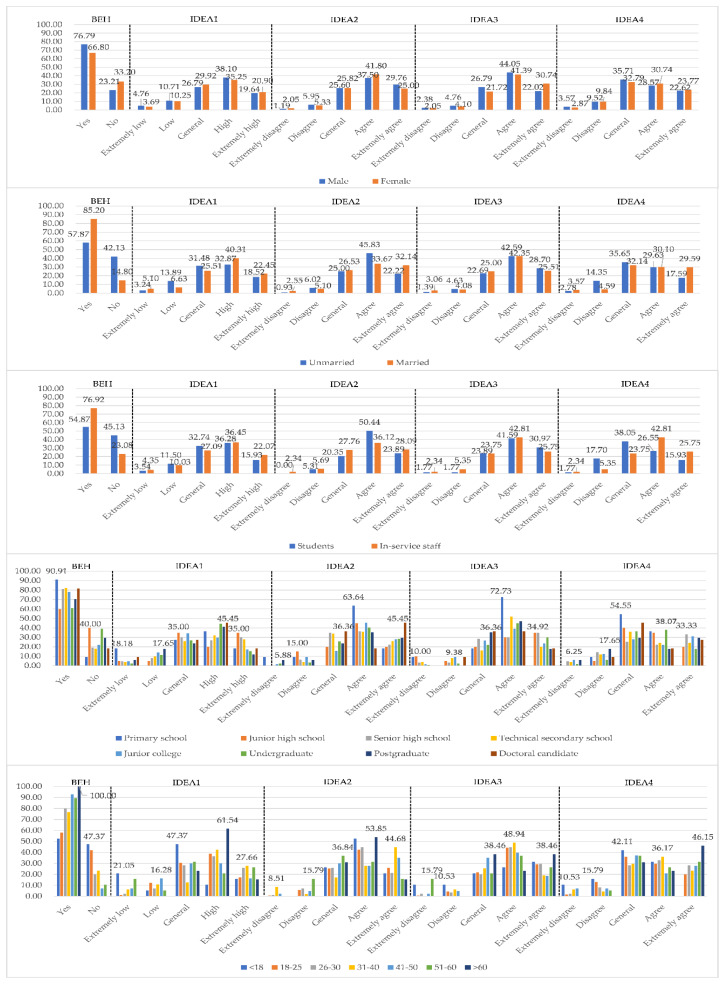
Group description. Note 1. BEH = Go out to rural tourism to relax in the last month; IDEA1 = Willingness to go out; IDEA2 = Willingness to choose a place close to home; IDEA3 = Traveling in countryside can relax the mind; IDEA4 = Rural tourism is safer. Note 2. The unit of data in the figure was “%”. Note 3. In the group description of education and age, the data label was indicated by the maximum value of that option.

Among the recovered samples, 244 were female, accounting for 59.22%. The sample covered all age groups, with 45.15% of respondents aged 18–25 and 52.43% unmarried, representing 113 full-time students. All of the respondents had attained a primary school degree or above, with the largest proportion of respondents having a bachelor’s degree (42.72%). Apart from the characteristics of demographic information, the recent willingness of respondents to go out and their basic viewpoints on rural tourism were also surveyed. Sample data distribution can be found in Table 3 and Figure 3.

As China was the first to be hit by the outbreak of pneumonia, it started to enforce comprehensive containment measures in February 2020. After two months (eight weeks) of intensive efforts, the national epidemic ended up being brought under control in late March. Therefore, the situation of the respondents was investigated for nearly a month. As shown in Table 3, 70.87% of the respondents admitted to the need for relaxation and had the experience of travelling to the countryside last month. In the meantime, a vast majority of the population showed a strong willingness to go out and choose a place close to where they live for relaxation, while only 60 samples showed a weak willingness to go out. To further analyze the differences in demographic information, Figure 3 was drawn.

Judging from the outings of the past month, the proportion of men (76.79%), married people (85.2%), and working people (76.92%) were higher than women (66.80%), unmarried people (57.87%) and students (54.87%) respectively. In terms of education level, the proportion of junior high school diploma and undergraduate students going out were lower, with 60% and 60.8% respectively. The distribution of going out by age increased in a stepwise manner. With the growth of age, the probability of going out to relax was higher. For item IDEA1-3, the answers focused on the “High/Agree” level. In addition to those people younger than 18 or older than 60, women (86.07 percent), people aged 26–30 (90.59%), doctoral candidates (90.91%), married people (88.27%), and employed people (85.62%) were more likely to go out. The willingness of men (92.86%), people aged 18–25 (93.55%), doctoral candidates (100%), unmarried people (93.06%), and students (94.69%) to choose rural tourism as a travel method was higher than relative control group. More women (93.85%), people aged 18–25 (95.16%), postgraduate level (100%), married (93.98%), and students (96.46%) admitted that traveling in the countryside can relax them. Different from the previous three questions, a higher proportion of people hold a negative attitude towards the safety of rural tourism. However, compared with other forms of tourism, 92.45 percent and 87.13 percent of the respondents believed that rural tourism is effective in relaxing mood while ensuring personal safety. Men (86.90%), those aged 31–50 (93.32%), those with a junior high school diploma (95%), those married (91.84%), and those with a job (92.31%) were more likely to say that rural tourism is indeed relatively safe. As revealed by the descriptive statistics, people were more optimistic about rural tourism.

#### 3.3.2. Data of the Items in Research Model

**Figure 4:**
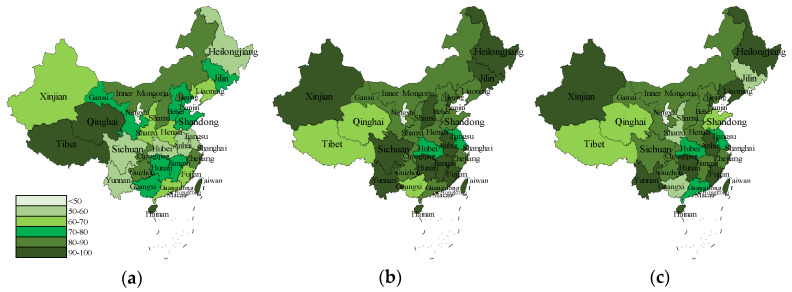
Distribution of travel and willingness to participate in rural tourism. Note 1. (**a**) The percentage distribution of people who chose “YES” to question BEH, “Go out to rural tourism to relax in the last month”. (**b**) The percentage distribution of people whose average travel intention was greater than 3. The average was calculated from the terms TI1-TI3. (**c**) The percentage distribution of people whose average travel intention was greater than 3. The average was calculated from the terms RI1-RI3. Note 2. Measures for all items were assessed with a five-point Likert scale from “Extremely unlikely/disagree/little/unacceptable “(1) to “Extremely likely/agree/well/acceptable” (5). Note 3. The unit of data in the figure was “%”.

In order to intuitively reflect regional Chinese residents’ views on rural tourism, a relevant transformation was carried out of the data from the items (BEH, TI1-TI3, RI1-RI3) to obtain Figure 3 and draw a comparison. The residents in most areas shared the experience of traveling to the countryside last month (Figure 4a) with a stronger desire (Figure 4b,c) to do so. The residents in Qinghai, Tibet and Guangxi province showed lower travel intention with regard to rural tourism, while the same situation happened with recommendation intention. The geographical distribution of travel intention and recommendation intention showed little variation; only the residents in Shanxi, Shandong and Jilin province had a lower willingness to recommend.

Under the two-factor model of perceived risk [99], each respondent was also required to rate the severity of each risk item presented in the questionnaire interview. Therefore, the square root value of probability of rural tourism risk occurrence and the severity of consequence were applied for the data on perceived risk in the model.

**Figure 5:**
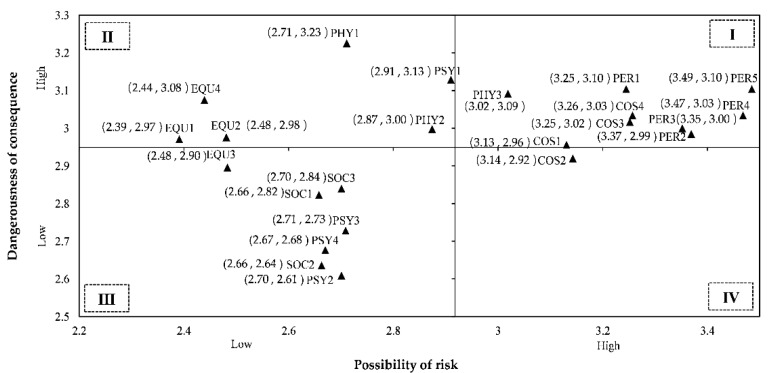
Quadrant distribution of perceived risk possibility and dangerousness of consequence. Note 1. PHY = Physical risk, EQU = Equipment risk, COS = Cost risk, PSY = Psychological risk, SOC = Social risk, PER = Performance risk. Note 2. The average values were in parentheses.

A quadrant diagram was used to visualize the distribution of perceived risk in all dimensions, as shown in Figure 5. In this paper, the average value of rural tourism risk possibility and the average severity were determined by the intersection point (2.949, 2.918) of the *X* axis and *Y* axis.

As shown in Figure 5, the items of time cost (COS3, COS4) and performance risk (PER1-5) were placed in the I quadrant, suggesting that potential tourists attach greater importance to time cost in rural tourism and the performance demands of the travel. Potential tourists during the epidemic were also very sensitive: “I may get sick on the trip. For example, getting pneumonia.” (PHY3). The II quadrant of the grid focused on two other items of physical risk (PHY1, PHY2), as well as most of the equipment risk items (EQU1, EQU2, EQU4). Despite the lower likelihood of violence and social security incidents at rural tourist attractions, any occurrence of such an incident would cause severe consequences. It was found out that with regard to rural tourism travel, in addition to PSY1, the rest of the psychological risk and all social risk items were distributed in the III quadrant, suggesting that current rural tourism social and psychological risk were subject to impact from the outbreak of COVID-19 pandemic, and that the risk of psychological damage was not deemed high among tourists. Finally, only one term (COS2) was observed at IV quadrant and item COS1 was situated at the intersection of the I quadrant and the IV quadrant. Despite the greater importance to tourists of time cost in rural tourism rather than economic cost, the probabilities of “Actual travel costs will exceed expectations during trip.” (COS1) and “I will spend undeserved money on rural tourism.” (COS2) were also high with limited loss.

## 4. Hypothesis Testing and Result Discussion

Over the course of research, IBM SPSS software was applied to analyze the reliability of the questions, while AMOS22 software was used to conduct the confirmatory factor analysis (CFA) and make result estimation from the structural equation model (SEM). With regard to questionnaire design, the questions were set for each dimension of rural tourism risk perception. As there were six dimensions involved in tourism risk perception (PCP), a second-order structural equation model was constructed to validate the correlation between variables for simplifying the model and estimate parameters.

### 4.1. Reliability and Validity Test

Firstly, a reliability analysis was carried out on the corresponding latent variables. The minimum Cronbach’s value of the 11 dimensions was 0.816, higher than the critical value of 0.7. In confirmatory factor analysis, the model fitting indexes of confirmatory factor analysis results also met the minimum requirements (χ2 = 1363.846, df = 764, *p* < 0.001, χ2/df = 1.785, RMSEA = 0.044, CFI = 0.941, IFI = 0.941, TLI = 0.933). Composite Reliability (CR) values of the scale were similar to the Cronbach’s coefficient values, and the Average Variance Extracted (AVE) of all dimensions exceeded 0.5 in Table 4, suggesting that the observed variables in the scale could have a significant impact on the corresponding latent variables. Moreover, the aggregate validity of each latent variable was excellent.

In addition to ensuring the convergent validity of various latent variables, the validity among the latent variables was also distinguished in Table 5. The square roots of AVE values were higher than standardized correlation coefficients with other latent variables. Latent variables were shown to have a good discriminant validity.

### 4.2. Structural Equation Model Analysis

**Figure 6:**
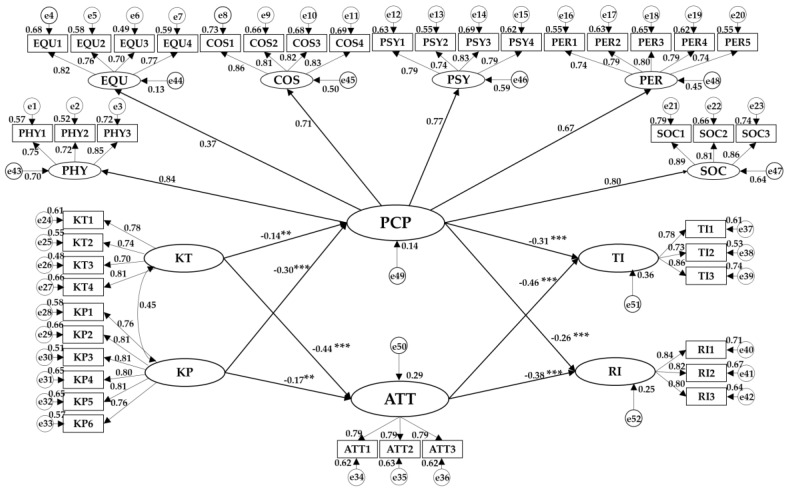
Structure Equation Model Diagram. Note 1. TI = Travel Intention, RI = Recommend Intention, KP = Knowledge of Pneumonia, KT = Knowledge of Tourism, ATT = Attitude to risk aversion, PCP = Perception of risk, PHY = Physical risk, EQU = Equipment risk, COS = Cost risk, PSY = Psychological risk, SOC = Social risk, PER = Performance risk.

Table 6 and Figure 6 present the model and estimation results of the second-order structural equation developed in this paper. The model fitting indexes were only slightly higher compared to the critical value, which is because the second-order model will reduce the model estimation parameters and cause the model fitting degree to decline. Table 6 indicates eight paths in the structural equation model, respectively. It can be seen clearly that the path coefficients were invariably negative and significant at 5% level. Therefore, H1-H6 and H11-H12 were verified by empirical research.

Based on the estimation results obtained from the structural model, tourism risk knowledge (KT) and pneumonia risk knowledge (KP) caused a negative impact on the risk perception (PCP) of rural tourism ($\beta_{\mathrm{KT}-\mathrm{PCP}}=-0.134,\;p\;<\;0.05;\;\beta_{\mathrm{KP}-\mathrm{PCP}}=-0.297,\;p\;<\;0.01$), which suggests that the greater the risk knowledge, the lower the risk perception of rural tourism [100]. Similarly, the richer the risk knowledge, the more the tourists prefer the rural tourism risk ($\beta_{\mathrm{KT}-\mathrm{ATT}}=-0.441,\;p\;<\;0.01;\;\beta_{\mathrm{KP}-\mathrm{ATT}}=-0.175,\;p\;<\;0.05$). By comparing the coefficient values of tourism risk knowledge (KT) with pneumonia risk knowledge (KP), it can be known that in the current epidemic stage, pneumonia risk knowledge had a more considerable effect on rural tourism risk perception (PCP), while rural tourism risk knowledge (KT) caused a more significant impact on rural tourism risk aversion attitude (ATT), which implies that the degree of knowledge of pneumonia risk is effective in alleviating the risk perception of potential tourists before their travel. Moreover, pneumonia risk knowledge exerted a less significant influence on the original risk aversion attitude to rural tourism, thus reflecting the inherent risk selection attribute [66].

As for travel intention (TI) and recommendation intention (RI) for rural tourism, the risk perception (PCP) and risk aversion attitude (ATT) of rural tourism both caused a significant negative impact ($\beta_{\mathrm{PCP}-\mathrm{TI}}=-0.289,\;p\;<\;0.01;\;\beta_{\mathrm{ATT}-\mathrm{TI}}=-0.417,\;p\;<\;0.01;\;\beta_{\mathrm{PCP}-\mathrm{RI}}=-0.311,\;p\;<\;0.01;\;\beta_{\mathrm{ATT}-\mathrm{RI}}=-0.436,\;p\;<\;0.01$), which is consistent with the theoretical hypothesis. When the risk perception of an individual is higher and he or she tends to avoid risks more, corresponding behavioral willingness will be lower. As revealed by the comparison between the coefficient values of risk perception (PCP) and risk aversion attitude (ATT) in rural tourism, risk aversion attitude (ATT) could have a more significant impact on behavioral intention, indicating that risk aversion attitude (ATT) plays a more significant role in deciding whether to go on travel or recommend rural tourism. The risk perception (PCP) and risk aversion attitude (ATT) of potential tourists for rural tourism exerted a greater influence on the recommendation intention compared to their own travel intention. When faced with a risky factor, people lack willingness to let others take risks.

### 4.3. Analysis of Mediating Effect

With the focus placed on the mediating effect of risk perception (PCP) and risk aversion attitude (ATT) in the model of rural tourism, the mediating effect of single path and parallel path was verified in this paper, with the verification results detailed in Table 7.

The mediating effect results of eight single paths and four parallel mediating paths are shown in Table 6. Of the 12 paths, what was insignificant at the significance level of 5% includes only the path coefficients of “TI < -- PCP < -- KT” and “RI < -- PCP < -- KT”. Though the relative confidence intervals passed 0, they were significant at the level of 10%, indicating the absence of a single mediating effect caused by rural tourism risk perception (PCP) on the correlation between tourism risk knowledge (KT) and behavioral intention towards rural tourism. Therefore, H7 and H8 were identified as invalid, which is because rural tourism is relatively safe, and a rich knowledge of tourism risks makes no significant change to perception. The confidence intervals of the other six single paths failed to pass 0, suggesting that rural tourism risk perception (PCP) acts as an intermediary in the correlation between pneumonia risk knowledge (KP), rural tourism travel intention (TI) and rural tourism recommendation intention (RI). The relationship of risk knowledge and behavioral intention towards rural tourism can be mediated by risk aversion attitude (ATT). Therefore, H9, H10, and H13-H16 were supported. When the path included risk perception and risk aversion attitude simultaneously, the parallel mediation effects were drawn out to verify the effectiveness of the rural tourism risk perception (PCP) involved in the model. All of the mediation effects passed the test of significance of 1%, suggesting the necessity that both risk perception and risk aversion attitude are taken into consideration in rural tourism. Thus, the structural equation model established in this paper was verified as reasonable.

## 5. Discussion and Implications

The degree of understanding as to risk can reduce perception of risk [39], thus influencing behavioral willingness [59]. However, the influence relationship showed different results depending on the exact scenario. In order for rural tourism managers to work out appropriate solutions, it is necessary to understand the relationship between current risk knowledge and the behavioral intention towards rural tourism, as well as the focus of potential tourists on the risks of rural tourism. Under the context of the Covid-19 epidemic, a structural model was constructed in this paper that involves tourism risk knowledge, pneumonia risk knowledge, risk perception, risk aversion attitude, travel intention and recommend intention. An analysis was conducted of relative relationships. As revealed by our empirical results, pneumonia risk knowledge can influence behavioral willingness to accept rural tourism.

According to the descriptive statistical analysis, most Chinese people showed preference for rural tourism as a way to relax over the weekend. Men and married people had a higher need to go out. The distribution of each dimension of perceived risk was analyzed, so as to clearly understand the perception differences of potential tourists in each dimension of perceived risk and provide reference for rural tourists. The results demonstrated that potential tourists pay more attention to the performance realization and time cost of rural tourism rather than the social-psychosocial factors. In the meantime, they believed that personal safety, or equipment and facilities problems were less likely to arise but could have a serious consequence. Therefore, as a manager of rural tourism, it is necessary to enhance the characteristics of scenic spots and improve the quality of scenic spots for meeting the functional needs of tourists and reducing customers’ perception of time risk. Additionally, the social security of scenic spots needs to be ensured, and the facilities construction at scenic spots ought to be improved for preventing the occurrence of personal safety problems. Faced with the current psychological fluctuations of tourists, stability of the epidemic situation is prioritized while controlling epidemic prevention efforts of the scenic spots. With regard to economic cost, the rural tourism managers need to ensure the reasonable pricing of products, improve the quality of scenic spots and mitigate the risk perception of economic cost to be burdened by tourists. When all the work is ready, oral communication will also bring tourists to scenic spots.

In the empirical part of the study, risk knowledge was negatively correlated with risk perception and risk aversion attitude. Rural tourism risk perception was primarily affected by pneumonia risk knowledge ($\beta_{\mathrm{KP}-\mathrm{PCP}}=-0.297,\;p\;<\;0.01$), while attitude to risk aversion was mainly influenced by tourism knowledge of risk ($\beta_{\mathrm{KT}-\mathrm{ATT}}=-0.441,\;p\;<\;0.01$), which reflected the volatility of risk perception and the inherent nature of risk aversion attitude to rural tourism. Among the impacts on the behavioral intention of rural tourism, the path coefficient values of risk aversion attitude were found to be more significant ($\beta_{\mathrm{PCP}-\mathrm{TI}}=-0.289,\;p\;<\;0.01;\;\beta_{\mathrm{ATT}-\mathrm{TI}}=-0.417,\;p\;<\;0.01;\;\beta_{\mathrm{PCP}-\mathrm{RI}}=-0.311,\;p\;<\;0.01;\;\beta_{\mathrm{ATT}-\mathrm{RI}}=-0.436,\;p\;<\;0.01$). Combined with the results of the mediation effect test, pneumonia risk knowledge caused a less significant but positive impact on the behavioral intention towards rural tourism than tourism risk knowledge, suggesting that tourists believed that the epidemic factors had been brought under control and won’t cause a major impact when considering traveling to the countryside. This confirmed that rural tourism was regarded as a better travel option amid the epidemic control phase. Combined with the above points, safer rural tourism in China indeed meets development opportunities during COVID-19 containment.

There are few empirical analyses focusing on the relationship between public health crisis knowledge and consumer behavior in existing literature, which were usually represented as descriptive statistical analyses. Considering the characteristics of epidemic-caused crisis, a model of the impact made by epidemic risk knowledge on the behavioral intention towards rural tourism was constructed in this paper from the perspective of perceived risk. Empirical analysis was carried out to validate the promotion effect of pneumonia risk knowledge on behavioral intention towards rural tourism, despite that it was not most important in this scenario. Therefore, while using media to spread information on tourist sites, tourism managers can also add information on epidemic prevention measures that tourists should pay attention to when visiting tourist sites and the relevant safety information at tourist sites, to increase the knowledge of potential tourists about the epidemic and reducing the risks perceived by tourists.

Risk perception and risk aversion attitude were frequently regarded as the major influencing factors in behavioral intention. However, the parallel mediating effect of the two have attracted little attention. Researches into the influence of risk knowledge on risk perception and the influence of risk knowledge on risk aversion attitude were carried out separately. Based on the logic of the existing literature, a parallel mediation model that involves risk perception and risk aversion attitude was constructed in this paper, and then the relevant hypotheses were verified. To some extent, this research provided feasible and basic consumer behavior research in different contexts.

## 6. Conclusions

With the “risk knowledge-risk perception-behavior intention” model and “risk knowledge-risk aversion attitude-behavior intention” model applied, our study tried to verify the impact of pneumonia risk knowledge on rural tourism willingness. 412 valid samples were collected by accidental sampling and snowball sampling method and the negative correlations between risk knowledge and risk perception towards rural tourism, risk knowledge and risk aversion attitude, risk perception and behavioral intentions of rural tourism, risk aversion attitude and behavioral intentions of rural tourism were proved by the SEM method. The research model in this paper involved 16 hypotheses, 14 of which were supported. It demonstrated that risk knowledge can reinforce behavioral intention through risk perception and risk attitude in the face of public health emergencies. The model established in this paper was identified as reasonable. Besides, the intermediary roles of rural tourism risk perception and risk aversion attitude were supported. The parallel mediating effect of risk perception and risk aversion attitude in rural tourism shall be taken into consideration together. Up to now, our findings have attempted to fill the research gap in consumer behavior under incomplete circumvention and public health emergencies to some extent and this was achieved in the field of rural tourism.

## 7. Limitations

In this paper, Chinese residents were chosen as the research object, so the universality of the results needs to be improved. Other influence factors were not considered in rural tourism such as tourism risk knowledge source, media coverage, cultural and political factors. Meanwhile, the information of respondents was collected only through online questionnaires and telephone interviews since face-to-face interviews cannot be conducted during the epidemic period, which restricted sample collection. Therefore, future research is proposed to enrich the research model, expand the sample size and enhance the representativeness of the research samples required to be improved.

## Figures and Tables

**Table 1:** Dimensions of tourism risk perception from research.

Researchers	Time	PHY	PER	PSY	SOC	FIN	TIM	EQU
Cheron and Ritchie [38]	1982	√	√	√	√	√	√	
Moutinho [16]	1987	√		√		√		
Verhage et al. [39]	1990	√	√		√	√	√	
Roehl et al. [7]	1992	√	√	√	√		√	√
Tsaur et al. [36]	1997	√	√		√			√
Sonmez and Graefe [40]	1998	√	√	√	√	√	√	√
Lepp and Gibson [26]	2003	√	√		√			
Dolnicar [41]	2005	√	√		√	√	√	
Reisinger and Mavondo [42]	2005	√	√	√	√	√	√	√
Han [43]	2005	√	√	√	√			√
Boksbergera et al. [44]	2007	√	√	√	√	√	√	
Liu and Gao [45]	2008	√	√	√	√	√		√
Qi et al. [46]	2009	√	√	√				
Quintal et al. [47]	2010	√	√	√	√	√		
Hu [48]	2011	√	√	√	√	√	√	
Fuchs and Reichel [49]	2011	√	√	√		√		
Zhu [50]	2013	√	√	√				
Xu et al. [34]	2013	√	√	√	√	√		√
Zhang and Yu [35]	2017	√	√	√		√	√	
Mohseni et al. [51]	2018	√	√			√	√	√
Xu et al. [52]	2019	√		√	√	√	√	
Yao and Hou [27]	2019	√	√	√	√	√		√

PHY = physical risk, PER = performance risk, PSY = psychological risk, SOC = social risk, FIN = financial risk, TIM = time risk, EQU = Equipment risk.

**Table 2:** Questionnaire items.

Variable		Item	Reference
TI		TI1: I would like to travel to the countryside for some time in the future.	Xu et al. [52], Lai and Chen [95], Zhao et al. [96]
		TI2: I prefer to travel to the countryside compared with other forms of tourism.	
		TI3: In the future, the feasibility of rural tourism will be high.	
RI		RI1: I will recommend rural tourism to a relative or friend.	
		RI2: I will introduce the advantages of rural tourism to relatives and friends.	
		RI3: When other people question me about outdoor leisure and relaxation, I will recommend rural tourism.	
KP		KP1: I know about the initial cause of COVID-19.	Li [6], Xu [52] Liu [55] Wang et al. [56]
		KP2: I know about the harm caused by COVID-19.	
		KP3: I know about the length of incubation period of COVID-19.	
		KP4: I know about the current affected range of COVID-19.	
		KP5: I know about the surveillance and warning signs for COVID-19.	
		KP6: I know about the preventive measures for COVID-19.	
KT		KT1: I am concerned about travel information.	
		KT2: I know about the causes of tourism risks.	
		KT3: I know about the consequences of tourism risks.	
		KT4: I know about the solutions to tourism risks.	
ATT		ATT1: I cannot accept going to travel to the countryside with family and friends.	Liu et al. [83], Zhang and Yu [35]
		ATT2: I cannot accept that local friends and relatives travel to the countryside.	
		ATT3: I will not eat with local friends and relatives after their trip to the countryside.	
PCP	PHY	PHY1: Man-made violent events, earthquakes, tsunamis, and other natural disasters may happen in rural tourism spots.	Han [43], Xu et al. [34], Yao and Hou [27], Zhang and Yu [35]
		PHY2: Public security incidents may occur at tourist sites.	
		PHY3: I may get sick on the trip, for example, pneumonia.	
	EQU	EQU1: Rural tourist attractions have poor infrastructure.	
		EQU2: Rural tourist attractions have poor sanitation.	
		EQU3: Traffic is inconvenient at rural tourist spots.	
		EQU4: Communication signal is poor at tourist sites.	
	COS	COS1: Actual travel costs will exceed expectations during a trip.	
		COS2: The money spent on rural tourism might be not worthy.	
		COS3: It takes plenty of time to plan and implement a rural tour.	
		COS4: Rural tourism will waste much time on the road.	
	PSY	PSY1: When considering rural tourism, I feel worried.	
		PSY2: I feel sick about engaging in rural tourism.	
		PSY3: I feel anxiety about engaging in rural tourism.	
		PSY4: I feel nervous about engaging in rural tourism.	
	SOC	SOC1: Other people will think negatively of me if I have a trip to rural spots.	
		SOC2: Going to rural tourism sites will make others criticize me.	
		SOC3: Friends and family member will not support my trip to the countryside	
	PER	PER1: At rural tourisms spots, food and entertainment arrangements are not as expected.	
		PER2: The appreciation of natural scenery and landscape are unsatisfactory.	
		PER3: Travel photography is not good at rural tourist sites.	
		PER4: Rural tourism does not enhance family bonds.	
		PER5: Rural tourism is unable to meet the requirements of relaxation.	

TI = Travel Intention, RI = Recommend Intention, KP = Knowledge of Pneumonia, KT = Knowledge of Tourism, ATT = Attitude of risk aversion, PCP = Perception of risk, PHY = Physical risk, EQU = Equipment risk, COS = Cost risk, PSY = Psychological risk, SOC= Social risk, PER = Performance risk. Measures for all items were assessed with a five-point Likert scale from “Extremely unlikely/disagree/little/unacceptable “(1) to “Extremely likely/agree/well/acceptable” (5). Table 2 shows the formal questionnaire for this paper, with the questionnaire items either deleted or adjusted after several rounds of preliminary research. Due to space limits, the relevant contents of the pre-survey were not presented in this paper.

**Table 3:** Descriptive statistics.

Items	Options	Sample (Percentage)	Items	Options	Sample (Percentage)
Gender	Male	168 (40.78%)	BEH	Yes	292 (70.87%)
	Female	244 (59.22%)		No	120 (29.13%)
Age	<18	19 (4.61%)	IDEA1	Extremely low	17 (4.13%)
	18–25	186 (45.15%)		Low	43 (10.44%)
	26–30	85 (20.63%)		General	118 (28.64%)
	31–40	47 (11.41%)		High	150 (36.41%)
	41–50	43 (10.44%)		Extremely high	84 (20.39%)
	51–60	19 (4.61%)	IDEA2	Extremely disagree	7 (1.70%)
	>60	13 (3.16%)		Disagree	23 (5.58%)
Education level	Illiteracy	0 (0.00%)		General	106 (25.73%)
	Primary school	11 (2.67%)		Agree	165 (40.05%)
	Junior high school	20 (4.85%)		Extremely agree	111 (26.94%)
	Senior high school	63 (15.29%)	IDEA3	Extremely disagree	9 (2.18%)
	Technical secondary school	50 (12.14%)		Disagree	18 (4.37%)
	Junior college	64 (15.53%)		General	98 (23.79%)
	Undergraduate	176 (42.72%)		Agree	175 (42.48%)
	Postgraduate	17 (4.13%)		Extremely agree	112 (27.18%)
	Doctoral candidate	11 (2.67%)	IDEA4	Extremely disagree	13 (3.16%)
Marital status	Unmarried	216 (52.43%)		Disagree	40 (9.71%)
	Married	196 (47.57%)		General	140 (33.98%)
Occupation	Students	113 (27.43%)		Agree	123 (29.85%)
	In-service staff	299 (72.57%)		Extremely agree	96 (23.30%)

BEH = Go out to rural tourism to relax in the last month; IDEA1 = Willingness to go out; IDEA2 = Willingness to choose a place close to home; IDEA3 = Traveling in countryside can relax the mind; IDEA4 = Rural tourism is safer.

**Table 4:** Data quality.

Item	Mean	Std.	Alpha.	CR	AVE	Item	Mean	Std.	Alpha.	CR	AVE
TI1	3.786	0.791	0.834	0.836	0.630	ATT1	2.442	0.788	0.839	0.840	0.636
TI2	3.840	0.736				ATT2	2.488	0.795			
TI3	3.840	0.850				ATT3	2.553	0.810			
RI1	3.607	0.837	0.861	0.861	0.673	PHY1	2.862	0.730	0.816	0.817	0.599
RI2	3.631	0.826				PHY2	2.868	0.718			
RI3	3.663	0.798				PHY3	2.988	0.866			
KP1	3.609	0.755	0.900	0.901	0.602	EQU1	2.569	0.830	0.847	0.848	0.583
KP2	3.544	0.818				EQU2	2.614	0.767			
KP3	3.636	0.710				EQU3	2.580	0.708			
KP4	3.488	0.802				EQU4	2.629	0.744			
KP5	3.893	0.803				COS1	2.977	0.855	0.899	0.899	0.691
KP6	4.049	0.763				COS2	2.952	0.811			
KT1	4.044	0.778	0.840	0.844	0.576	COS3	3.056	0.823			
KT2	4.061	0.745				COS4	3.073	0.835			
KT3	3.898	0.698				PSY1	2.957	0.777	0.868	0.869	0.625
KT4	3.976	0.810				PSY2	2.590	0.751			
PER1	3.103	0.728	0.881	0.882	0.598	PSY3	2.656	0.839			
PER2	3.096	0.792				PSY4	2.608	0.792			
PER3	3.104	0.806				SOC1	2.668	0.888	0.890	0.891	0.731
PER4	3.163	0.792				SOC2	2.585	0.816			
PER5	3.210	0.747				SOC3	2.704	0.859			

Std. = Standardized Loading, Alpha. = Cronbach Alphas, CR = Composite Reliability, AVE = Average Variance Extracted. TI = Travel Intention, RI = Recommend Intention, KP = Knowledge of Pneumonia, KT = Knowledge of Tourism, ATT = Attitude of risk aversion, PHY = Physical risk, EQU = Equipment risk, COS = Cost risk, PSY = Psychological risk, SOC = Social risk, PER = Performance risk. All standardized loadings were significant (*p* < 0.01).

**Table 5:** Discriminant validity.

	PER	SOC	PSY	COS	EQU	PHY	ATT	KT	KP	RI	TI
PER	0.774										
SOC	0.550	0.855									
PSY	0.368	0.737	0.790								
COS	0.602	0.522	0.501	0.831							
EQU	0.234	0.154	0.262	0.216	0.764						
PHY	0.650	0.635	0.600	0.620	0.314	0.774					
ATT	0.094	0.168	0.129	0.167	0.445	0.246	0.798				
KT	0.177	0.162	0.104	0.209	0.524	0.206	0.488	0.759			
KP	0.066	0.259	0.296	0.268	0.429	0.256	0.328	0.445	0.776		
RI	0.072	0.231	0.264	0.200	0.514	0.290	0.390	0.362	0.418	0.820	
TI	0.173	0.273	0.368	0.191	0.555	0.315	0.480	0.469	0.416	0.514	0.794

TI = Travel Intention, RI = Recommend Intention, KP = Knowledge of Pneumonia, KT = Knowledge of Tourism, ATT = Attitude of risk aversion, PHY = Physical risk, EQU = Equipment risk, COS = Cost risk, PSY = Psychological risk, SOC = Social risk, PER = Performance risk.

**Table 6:** Path analysis results.

Links		Unstd.	Std.	S.E.	*t*-Value
PCP < ---KT		−0.134	−0.139	0.062	−2.184 **
PCP < ---KP		−0.297	−0.296	0.065	−4.587 ***
ATT < ---KT		−0.441	−0.444	0.063	−6.952 ***
ATT < ---KP		−0.175	−0.17	0.06	−2.916 **
TI < ---PCP		−0.289	−0.311	0.052	−5.541 ***
TI < ---ATT		−0.417	−0.461	0.052	−7.967 ***
RI < ---PCP		−0.311	−0.264	0.066	−4.692 ***
RI < ---ATT		−0.436	−0.379	0.065	−6.682 ***
**Fitness** **Index**	**Statistics**	**Critical** **Value**	**Fitness** **Index**	**Statistics**	**Critical** **Value**
x2	1739.848	the smaller, the better	CFI	0.908	>0.900
x2/df	2.164	[1,3]	IFI	0.908	>0.900
RMSEA	0.053	<0.080	TLI	0.901	>0.900

Unstd. = unstandardized coefficient, Std. = standardized coefficient. TI = Travel Intention, RI = Recommend Intention, KP = Knowledge of Pneumonia, KT = Knowledge of Tourism, ATT = Attitude to risk aversion, PCP = Perception of risk. ** *p* < 0.05, *** *p* < 0.01.

**Table 7:** Results of mediation effect test.

Links	Estimate	S.E.	*t*-Value	95% Confidence Intervals
TI < ---PCP < ---KT	0.046	0.028	1.653	[0.001, 0.107]
TI < ---ATT < ---KT	0.216	0.051	4.266	[0.126, 0.324]
RI < ---PCP < ---KT	0.049	0.030	1.622	[0.001, 0.116]
RI < ---ATT < ---KT	0.225	0.059	3.844	[0.123, 0.352]
TI < ---PCP < ---KP	0.089	0.029	3.121	[0.040, 0.153]
TI < ---ATT < ---KP	0.076	0.037	2.027	[0.010, 0.158]
RI < ---PCP < ---KP	0.096	0.035	2.784	[0.038, 0.175]
RI < ---ATT < ---KP	0.142	0.044	3.208	[0.067, 0.241]
TI < ---PCP and ATT < ---KT	0.261	0.053	4.972	[0.167, 0.374]
RI < ---PCP and ATT < ---KT	0.275	0.061	4.516	[0.166, 0.406]
TI < ---PCP and ATT < ---KP	0.165	0.047	3.530	[0.079, 0.263]
RI < ---PCP and ATT < ---KP	0.172	0.052	3.286	[0.079, 0.284]

TI = Travel Intention, RI = Recommend Intention, KP = Knowledge of Pneumonia, KT = Knowledge of Tourism, ATT = Attitude to risk aversion, PCP = Perception of risk.
